# Clinicopathological Characteristics of Thyroid Cancer: A 10-Year Experience at a Tertiary Care Center in Saudi Arabia

**DOI:** 10.7759/cureus.76068

**Published:** 2024-12-20

**Authors:** Awad Alshahrani, Sameerah Alshehri, Ibrahim Ajwah, Faisal Alotay, Faisal S Alqahtani

**Affiliations:** 1 Department of Medicine, College of Medicine, King Saud Bin Abdulaziz University for Health Sciences, Riyadh, SAU; 2 Department of Medicine, Ministry of National Guard-Health Affairs, Riyadh, SAU; 3 Department of Medicine, King Abdullah International Medical Research Center, Riyadh, SAU; 4 Department of Internal Medicine, King Salman Armed Forces Hospital, Tabuk, SAU; 5 College of Medicine, King Saud Bin Abdulaziz University for Health Sciences, Riyadh, SAU

**Keywords:** bethesda, fine-needle aspiration cytology, histopathology, malignancy, thyroid cancer

## Abstract

Background

Thyroid nodules are typically an initial sign of thyroid cancer (TC) and require evaluation by thyroid ultrasonography. Additional measures, such as fine needle aspiration, may be necessary depending on the level of malignancy risk. This study aims to comprehensively analyze TC clinical, radiological, and histopathological characteristics in a cohort of Saudi patients.

Methods

A retrospective chart review was carried out on TC patients who were being followed up at a tertiary care center in the Riyadh region of Saudi Arabia. The study included all patients aged 15 years and above diagnosed between 2013 and 2022 with adequate histopathological data. Statistical analysis used: SPSS v.27 was used for the data analysis.

Results

In total, 272 patients were included in the study, with a mean age of 47.23 ± 12.6 years. Of these, 222 (81.6%) were females. Nearly two-thirds of the affected patients had a body mass index (BMI) exceeding 25. The most common histological type of cancer was classical papillary thyroid carcinoma, accounting for 58.5% of cases. The average tumor size was 2.52 cm. The majority of patients presented with TNM stage I (86%), while one-fourth of the study population had thyroid imaging reporting and data systems (TI-RADS) 6 and Bethesda V. metastatic lymph nodes were predominantly among males (57.8%), and those younger than 50 years of age. The extrathyroidal extension was observed in 72% of tall-cell papillary thyroid carcinomas and 62.5% of oncocytic variant papillary thyroid carcinomas, compared to less than 50% in other subtypes (p < 0.001).

Conclusions

In our study, TC primarily affects obese middle-aged women. However, male gender and younger age are significant risk factors for lymph node involvement. Additionally, histopathological subtypes, rather than clinical parameters, are more likely to increase the risk of extrathyroidal extension. Given the conflicting data regarding clinical risk factors associated with the development of TC, lymph node metastasis, and extrathyroidal extension, further research is needed to clarify these discrepancies.

## Introduction

Thyroid cancer (TC) is the most common type of endocrine malignancy. It originates from the thyroid parenchyma, which contains two types of cells, namely thyroid follicular cells, which lead to differentiated thyroid cancer (DTC), and parafollicular cells (C cells), which lead to medullary thyroid carcinoma (MTC). DTC accounts for 90-95% of all thyroid malignancies, while MTC accounts for only 1-2%. Anaplastic thyroid carcinoma is the rarest type of TC, accounting for less than 1% of all TC [1].

The incidence of DTC has been steadily increasing at an annual rate of around 5%. This rise could be attributed to an increase in early diagnoses of subclinical cases of DTC through the more liberal use of advanced imaging technologies. Currently, it is estimated that approximately 40-50% of all papillary TCs are indolent, with limited clinical significance [2].

In the Surveillance, Epidemiology, and End Results (SEER) report, the incidence of papillary TC, which is the most common DTC type, increased from 4.8 to 14.9 per 100,000 population between 1975 and 2018. However, despite the increased incidence, the death rate has remained stable, estimated to be 0.5 per 100,000 population per year [3].

Similarly, in Saudi Arabia, a retrospective study by Hussain et al. reported an increase in DTC incidence throughout the Kingdom from 2000 to 2010 [4]. This increase in incidence was attributed to the increased use of neck ultrasonography and fine needle aspiration (FNA) of thyroid nodules, which have led to increased and earlier detection of small papillary TC [5].

However, a study conducted on the National Cancer Institute’s SEER-9 Cancer Registry Database suggested that there has been an increase in the rates of DTC of all sizes, including tumors larger than 4 cm. Therefore, although the reported epidemiological data are reassuring in terms of DTC survival, this will not necessarily continue to be the case due to the increased incidence of advanced tumors of this type [6,7].

DTCs are commonly diagnosed in the setting of thyroid nodules, which are a common clinical problem, affecting up to 60% of the general population. This has led to the development of radiological scores to identify thyroid nodules’ risk of being malignant. The thyroid imaging reporting and data system (TI-RADS) score is a validated and widely used ultrasound-based risk categorization of thyroid nodules.

DTC has many histopathological subtypes with variable clinical behaviors. Moreover, the histopathological features of DTC, such as the TNM stage, the multifocality of the tumor, and its extension to the adjacent structures, are important features in predicting the clinical course of a tumor [8].

There is limited research on the clinical and pathological characteristics of DTC in the Middle East region. This study aims to provide a comprehensive analysis of TC’s clinical, radiological, and histopathological characteristics in a cohort of Saudi patients. Determining the characteristics of TC among the Saudi population can improve our understanding of the effects of demographic factors on TC. It can also help identify positive and negative prognostic factors that impact patient outcomes.

## Materials and methods

The Ethics Committee of King Abdullah International Medical Research Center approved a retrospective analysis of the clinical, radiological, and pathological profiles of patients with TC who attended TC clinics at King Abdulaziz Medical City (KAMC) in Riyadh, Saudi Arabia between 2013 and 2022. The consent was taken from all the patients when they came to the hospital and clinics, their anonymous data will be used in the future for research purposes.

All adult patients diagnosed with TC based on the post-thyroidectomy surgical pathology results were included in the study. Adult patients with TC not diagnosed through the post-thyroidectomy surgical pathology were excluded.

Clinical, radiographic, and pathological data were collected from patients’ electronic medical records. These data included demographic and clinical variables related to gender, age, body mass index (BMI), sonographic characteristics, FNA cytology results, type of surgery, histopathological subtype, and TNM stage.

Thyroid ultrasounds were interpreted and reported by a board-certified radiologist using the TI-RADS 1-5 classification system of thyroid nodules, while thyroid cytology and pathology were interpreted and reported by a board-certified pathologist using the six-tiered diagnostic categories proposed by the Bethesda System for Reporting Thyroid Cytopathology (TBSRTC) [9].

In this study, percentages and proportions are used to present categorical variables, while mean and standard deviation are used to present numerical variables. We employed a chi-squared test to assess the association between factors such as the size of the tumor, tumor multifocality, extrathyroidal extension, lymphovascular invasion (LVI), and lymph node metastasis by final histopathology and tumor size, gender, or age group. For the inferential statistical tests, a p-value <0.05 was used as the cut-off value for statistical significance. SPSS v.27 was used for the data analysis.

## Results

Demographic and clinical profile of the thyroid carcinoma patients

Table 1 presents the demographic characteristics of the 272 patients diagnosed with TC during the study period. The mean age at diagnosis was 47.23 ± 12.6 years. Notably, 43.4% of the patients diagnosed were above the age of 50. Most (81.6%) were female. The mean BMI was 32.1 kg/m^2^, with a significant portion of patients (36.8%) experiencing class 1 obesity. Only 1.1% were underweight (Table 1).

**Table 1:** Characteristics of patients diagnosed with thyroid tumor (N = 272).

Characteristics	n (%)
Age at diagnosis, Mean ± SD	47.23 ± 12.6
Age group at diagnosis (years), n (%)	
15–30	39 (14.3)
31–50	115 (42.3)
>50 years	118 (43.4)
Gender, n (%)	
Male	50 (18.4)
Female	222 (81.6)
Body mass index, Mean ± SD	32.1 ± 5.53
BMI classification, n (%)	
Underweight (<18.5)	3.0 (1.1)
Healthy weight (18.5–24.9)	29.0 (10.7)
Overweight (25–29.9)	55.0 (20.2)
Class 1 obesity (30–34.9)	100.0 (36.8)
Class 2 obesity (35–39.9)	65.0 (23.9)
Class 3 obesity (>40)	20.0 (7.4)

The average tumor size was 2.52 cm, with the majority of patients having a tumor size between 0.1 and 0.9 cm and less than one-third (23.2%) having a tumor size between 1 and 1.9 cm. Around 11% of patients had a tumor size greater than 6 cm. Additionally, the tumor was multifocal in 58.8% of patients. Extrathyroidal extension and LVI were present in 26.1% and 17.3% of patients, respectively. Lymph node metastasis was absent in most patients (67.6%), and no patient had distant metastasis (Table 2).

**Table 2:** Clinical profile of thyroid carcinoma patients (N = 272).

Clinical characteristics	n (%)
Tumor size (cm), Mean ± SD	2.52 (2.07)
Tumor size (cm), n (%)	
0.1–0.9 cm	76 (27.9)
1.0–1.9 cm	63 (23.2)
2.0–2.9 cm	35 (12.9)
3.0–3.9 cm	34 (12.5)
4.0–4.9 cm	17 (6.3)
5.0–5.9 cm	17 (6.3)
≥6 cm	30 (11.0)
Tumor multifocality	
No	112 (41.2)
Yes	160 (58.8)
Extrathyroidal extension	
No	201 (73.9)
Yes	71 (26.1)
Lymphovascular invasion	
No	225 (82.7)
Yes	47 (17.3)
Lymph node metastasis	
No lymph node metastasis	184 (67.6)
Level 1 lymph node metastasis	21 (7.7)
Level 2 lymph node metastasis	8 (2.9)
Level 3 lymph node metastasis	9 (3.3)
Level 4 lymph node metastasis	13 (4.8)
Level 6 lymph node metastasis	37 (13.6)
Distant metastasis	
No	272 (100)
Yes	0 (0)
TNM stage	
I	234 (86)
II	30 (11)
III	4 (1.5)
IV	4 (1.5)
Tumor type	
Classical papillary thyroid cancer	159 (58.5)
Follicular variant papillary thyroid cancer	54 (19.9)
Tall-cell papillary thyroid cancer	25 (9.2)
Follicular thyroid cancer, minimally invasive	12 (4.4)
Oncocytic variant papillary thyroid carcinoma	8 (2.9)
Medullary	7 (2.6)
Hürthle cell cancer	4 (1.4)
Follicular thyroid cancer, widely invasive	3 (1.1)
Type of surgical thyroidectomy	
Total thyroidectomy	135 (49.6)
Total thyroidectomy + central neck dissection	67 (24.6)
Total thyroidectomy + lateral neck dissection	8 (2.9)
Total thyroidectomy + central + lateral neck dissection	43 (15.8)
Hemithyroidectomy	10 (3.7)
Lobectomy	9 (3.3)
Radioactive iodine therapy	
Not received	141 (52)
Received	131 (48)
Additional surgical interventions	
None	245 (90.1)
Additional surgery	27 (9.9)

The majority of patients (86%) were at TNM stage I and only a few (1.5%) were diagnosed with TNM stages III or IV. Classical papillary thyroid tumor was the most common type, followed by follicular variant papillary TC. Approximately 50% of patients had undergone total thyroidectomy, whereas around a quarter (24.6%) had had total thyroidectomy with central neck dissection. Additional surgery was performed on 9.9% of the patients, and radioactive iodine therapy was given to 48% (Table 2).

Distribution of thyroid imaging reporting and data systems (TI-RADS) categories according to fine needle aspiration (FNA) cytology results

TI-RADS reporting and FNA cytology results were available for 169 patients. The majority of patients had TI-RADS 4 and 5 nodules on the ultrasound (39.6% and 40.8%, respectively), while 13% had TI-RADS 3 and 4.8% had TI-RADS 2 (Table 3). FNA cytology results of Bethesda categories V and VI were present in the majority of patients (24.3% and 40.8%, respectively) (Table 3). Notably, only approximately 25% of patients were categorized as ultrasound TI-RADS category 5 and FNA Bethesda category VI, while no patient had a TI-RADS 5 nodule with FNA Bethesda I (Table 3).

**Table 3:** Distribution of TI-RADS categories according to FNA cytology results (Bethesda) TI-RADS: Thyroid Imaging Reporting and Data Systems; FNA: fine needle aspiration

	TI-RADS, n (%)					Total
FNA (Bethesda)	1	2	3	4	5	
I	0 (0)	0 (0)	0 (0)	1 (0.6)	0 (0)	1 (0.6)
II	2 (1.20)	2 (1.20)	7 (4.10)	12 (7.10)	0 (0)	23 (13.6)
III	1 (0.6)	1 (0.6)	3 (1.80)	10 (5.90)	7 (4.10)	22(13.0)
IV	0 (0)	0 (0)	3 (1.80)	7 (4.10)	3 (1.80)	13 (7.7)
V	0 (0)	5 (3.0)	9 (5.30)	10 (5.90)	17 (10.10)	41 (24.3)
VI	0 (0)	0 (0)	0 (0)	27 (16.0)	42 (24.90)	69 (40.8)
Total	3 (1.80)	8 (4.8)	22 (13.0)	67 (39.60)	69 (40.80)	169 (100)

Distribution of FNA cytology results (Bethesda) according to final histopathology

Table 4 shows the distribution of FNA cytology results (Bethesda) and the final histopathology of the tumor. Most patients with classical papillary TC had Bethesda V (16.2%) and VI cytology (26.6%). In contrast, most patients with a follicular variant of papillary TC had Bethesda II and III. There were only three patients with widely invasive follicular TC, and all had Bethesda IV cytology. All four patients with Hürthle cell cancer had Bethesda V (Table 4).

**Table 4:** Distribution of FNA cytology results (Bethesda) according to final histopathology, n (%). FNA: fine needle aspiration

FNA (Bethesda)	Determining the characteristics of thyroid cancer among the Saudi population can improve our understanding of the effects of demographic factors on TC. It can also help identify positive and negative prognostic factors that impact patient outcomes.							Total
	Classic Papillary Thyroid Cancer	Follicular variant papillary thyroid cancer	Tall-Cell Papillary Thyroid Cancer	Follicular thyroid cancer, widely invasive	Follicular thyroid cancer, minimally invasive	Hürthle cell cancer	Oncocytic variant papillary thyroid carcinoma	
I	0 (0)	0 (0)	0 (0)	0 (0)	1 (0.38)	0 (0)	0 (0)	1 (0.38)
II	14 (5.4)	17 (6.5)	0 (0)	0 (0)	2 (0.77)	0 (0)	2 (0.77)	35 (13.5)
III	24 (9.3)	18 (6.94)	2 (0.77)	0 (0)	2 (0.77)	0 (0)	0 (0)	46 (17.8)
IV	7 (2.7)	6 (2.32)	2 (0.77)	3 (1.15)	5 (1.93)	0 (0)	0 (0)	23 (8.9)
V	42 (16.22)	8 (3.08)	0 (0)	0 (0)	0 (0)	4 (1.59)	3 (1.16)	57 (22.0)
VI	69 (26.6)	5 (5.15)	20 (20.6)	0 (0)	0 (0)	0 (0)	3 (1.16)	97 (37.5)
Total	156 (60.23)	54 (20.8)	24 (9.26)	3 (1.16)	10 (3.86)	4 (1.59)	8 (3.08)	259 (100)

Distribution of TI-RADS categories according to final histopathology

Table 5 shows the distribution of the TI-RADS categories and the final histopathology of the tumor. About a quarter (21.17%) and one-third (33.5%) of patients with classical papillary TC were classified under TI-RADS 4 and TI-RADS5, respectively. All patients with tall-cell papillary TC; follicular TC, widely invasive; follicular TC, minimally invasive; Hürthle cell cancer, and oncocytic variant papillary thyroid carcinoma fell under either TI-RADS 4 or TI-RADS 5, as shown in Table 5.

**Table 5:** Distribution of TI-RADS categories according to final histopathology, n (%). TI-RADS: Thyroid Imaging Reporting and Data Systems

TI-RADS	Final histopathology (tumor type)							Total
	Classical papillary thyroid cancer	Follicular variant papillary thyroid cancer	Tall-cell papillary thyroid cancer	Follicular thyroid cancer, widely invasive	Follicular thyroid cancer, minimally invasive	Hürthle cell cancer	Oncocytic variant papillary thyroid carcinoma	
1	3 (1.76)	1 (0.59)	0 (0)	0 (0)	0 (0)	0 (0)	0 (0)	4 (2.4)
2	6 (3.53)	2 (1.17)	0 (0)	0 (0)	0 (0)	0 (0)	0 (0)	8 (4.7)
3	11 (6.47)	11 (6.47)	0 (0)	0 (0)	0 (0)	0 (0)	0 (0)	22 (12.9)
4	36 (21.17)	14 (8.23)	10 (5.88)	2 (1.17)	2 (1.17)	2 (1.17)	1 (0.59)	67 (39.4)
5	57 (33.5)	1 (0.59)	8 (4.70)	1 (0.59)	0 (0)	0 (0)	2 (1.17)	69 (40.6)
Total	113 (66.4)	29 (17.0)	18 (10.6)	3 (1.76)	2 (1.17)	2 (1.17)	3 (1.76)	170 (100.0)

Comparison of tumor size, tumor multifocality, extrathyroidal extension, lymphovascular invasion, and lymph node metastasis by age, gender, and final histopathology

Table 6 compares tumor size, multifocality, extrathyroidal extension, LVI, and lymph node metastasis by age and gender. Pearson Chi-Square test was used, which showed that around 75.6% of the male patients had tumor multifocality, compared to 56.4% of females (p = 0.02), suggesting a significant difference in tumor multifocality by gender. However, there was no difference in tumor focality by age group (p = 0.11). Similarly, a more significant proportion of males (57.8%) was found to have lymph node metastasis, compared to only 25% of females (p <0.001). Likewise, 36.7% of patients under 50 years of age significantly had shown lymph node metastasis, compared to 22.9% of patients aged over 50 years (p = 0.01). These findings suggest that lymph node metastasis differed by gender and age group. On the other hand, there were no significant differences in extrathyroidal extension and LVI either by gender or by age group, as shown in Table 6. 

**Table 6:** Comparison of tumor size, tumor multifocality, extrathyroidal extension, lymphovascular invasion, and lymph node metastasis by age and gender *Pearson Chi-Square test ** Correlation is significant at the 0.05 level (2-tailed).

Characteristic of tumor	Gender			Age group		
	Male	Female	p-value	≤50 years	>50 years	p-value*
Tumor size						
<1 cm	2 (4.4%)	12 (5.5%)	0.78	11 (7.5%)	3 (2.5%)	0.07
≥1 cm	43 (95.6%)	208 (94.5%)		136 (92.5%)	115 (97.5%)	
Tumor multifocality						
No	11 (24.4%)	96 (43.6%)	0.02**	53 (36.1%)	54 (45.8%)	0.11
Yes	34 (75.6%)	124 (56.4%)		94 (63.9%)	64 (54.2%)	
Extrathyroidal extension						
No	35 (77.8%)	164 (74.5%)	0.65	112 (76.2%)	87 (73.7%)	0.64
Yes	10 (22.2%)	56 (25.5%)		35 (23.8%)	31 (26.3%)	
Lymphovascular invasion						
No	41 (91.1%)	184 (83.6%)	0.20	130 (88.4%)	95 (80.5%)	0.07
Yes	4 (8.9%)	36 (16.4%)		17 (11.6%)	23 (19.5%)	
Lymph node metastasis						
No	19 (42.2%)	165 (75.0%)	<0.001**	93 (63.3%)	91 (77.1%)	0.01**
Yes	26 (57.8%)	55 (25.0%)		54 (36.7%)	27 (22.9%)	

Table 7 compares the final histopathology of tumor size, multifocality, extrathyroidal extension, LVI, and lymph node metastasis. The data revealed that across all histopathological subtypes, tumor size was significantly greater than 1 cm (p = 0.005). When comparing multifocality by tumor type, it is primarily observed in papillary TC. Multifocality occurs in 72% of tall-cell papillary TC and 63.5% of classic papillary TC. Moreover, 57.4% of the follicular variant papillary TC and 75% of the oncocytic variant papillary thyroid carcinomas were multifocal as well. All patients with follicular TC, widely and minimally invasive follicular TC, had a unifocal disease (p <0.001). With respect to extrathyroidal extension, 72% of tall-cell papillary TC and 62% of oncocytic variant papillary thyroid carcinomas showed extrathyroidal extension, compared to less than 50% of the other subtypes (p <0.001).

**Table 7:** Comparison of tumor size, tumor multifocality, extrathyroidal extension, lymphovascular invasion, and lymph node metastasis by age and gender (N=265) *Pearson Chi-Square test **Correlation is significant at the 0.05 level (2-tailed).

Characteristic of tumor	Final histopathology (tumor type)							p-value*
Tumor size	Classical papillary thyroid cancer	Follicular variant papillary thyroid cancer	Tall-cell papillary thyroid cancer	Follicular thyroid cancer, widely invasive	Follicular thyroid cancer, minimally invasive	Hürthle cell cancer	Oncocytic variant papillary thyroid carcinoma	
<1 cm	5 (3.1)	9 (16.7)	0 (0.0)	0 (0)	0 (0)	0 (0)	0 (0)	0.005**
≥1 cm	154 (96.9)	45 (83.3)	25 (100)	3 (100)	12 (100.0)	4 (100)	8 (100)	
Tumor multifocality								
No	58 (36.5)	23 (42.6)	7 (28.0)	3 (100)	12 (100.0)	2 (50)	2 (25)	<0.001**
Yes	101 (63.5)	31 (57.4)	18 (72.0)	0 (0)	0 (0)	2 (50)	6 (75)	
Extrathyroidal extension								
No	127 (79.9)	46 (85.2)	7 (28.0)	2 (66.7)	10 (83.3)	4 (100)	3 (37.5)	<0.001**
Yes	32 (20.1)	8 (14.8)	18 (72.0)	1 (33.3)	2 (16.7)	0 (0)	5 (62.5)	
Lymphovascular invasion								
No	139 (87.4)	54 (100)	15 (60.0)	3 (100)	9 (75.0)	0 (0)	5 (62.5)	<0.001**
Yes	20 (12.6)	0 (0)	10 (40.0)	0 (0)	3 (25)	4 (100)	3 (37.5)	
Lymph node metastasis								
No	98 (61.6)	48 (88.9)	11 (44)	3 (100)	12 (100)	4 (100)	8 (100)	<0.001**
Yes	61 (38.4)	6 (11.1)	14 (56.0)	0 (0)	0 (0)	0 (0)	0 (0)	

Regarding LVI, 100% of the Hürthle cell cancers showed LVI, compared to 37.5% and 12.6% of oncocytic variant papillary thyroid carcinomas and classical papillary TC, respectively (p <0.001).

Lymph node metastasis varies significantly according to the different TC subtypes. It is commonly observed in tall-cell papillary thyroid carcinoma (56%) and classic papillary thyroid carcinoma (38.4%). In contrast, only 11.1% of cases involving the follicular variant of papillary thyroid carcinoma show lymph node metastasis. No cases of lymph node metastasis were observed in the other subtypes (p < 0.001), as shown in Table 7.

Tumor multifocality, extrathyroidal extension, lymphovascular invasion, and lymph node metastasis according to tumor size

Table 8 provides insights into the relationship between tumor size and factors such as tumor multifocality, extrathyroidal extension, LVI, and lymph node metastasis. The data suggest there to be no significant correlation between tumor size and multifocality, as indicated by a p-value of 0.45. A higher percentage of tumors ≥1 cm (25.9%) had extrathyroidal extension than tumors <1 cm (7%), although this difference was not statistically significant (p = 0.11). Additionally, there were no significant differences in tumor size concerning LVI (p = 0.10) or lymph node metastasis (p = 0.17).

**Table 8:** Correlation between tumor size with tumor multifocality, extrathyroidal extension, lymphovascular invasion, and lymph node metastasis (N=265). *Pearson Chi-Square test

Characteristic of tumor	Tumor size		p-value*
	<1 cm	≥1 cm	
Tumor multifocality			
No	7 (50)	100 (39.8)	0.45
Yes	7 (50)	151 (60.2)	
Extrathyroidal extension			
No	13 (92.9)	186 (74.1)	0.11
Yes	1 (7.1)	65 (25.9)	
Lymphovascular invasion			
No	14 (100)	211 (84.1)	0.10
Yes	0 (0)	40 (15.9)	
Lymph node metastasis			
No	12 (85.7)	172 (68.5)	0.17
Yes	2 (14.3)	79 (31.5)	

## Discussion

The current study describes the demographic, radiological, and histological characteristics of patients who had undergone thyroidectomy for TC at KAMC in Riyadh, Saudi Arabia, between 2013 and 2022.

Our study included 272 patients diagnosed with TC, of whom 81.6% were females. In keeping with previous research results, females were more affected than males, at a ratio of 4.4:1.0. This higher rate of TC among females could be due to various causes, such as hormonal factors that accelerate the growth of microscopic tumors. Furthermore, the growth of the entire thyroid during pregnancy as a response to increased thyroid stimulating hormone and human chorionic gonadotropin may have played a role [10-12].

Most patients (43.4%) had been diagnosed at 50 years or older, contrary to the previously published study in which Karkuzhali et al. demonstrated that only 26.3% of patients had been diagnosed at the age of 45 years or older [13].

The study found that the median age of diagnosis was 49 years for females and 46 for males, higher than in previous studies. For example, the retrospective study conducted by Samargandy et al. in the Western region of Saudi Arabia reported a mean age of diagnosis of 40.6 years. A similar pattern was observed in the United Arab Emirates, where 71% of patients were under the age of 45. Data from the Saudi Cancer Registry show that the median age at diagnosis is 40 years for females and 45 years for males [14-16].

In contrast, a study in the United States found that of the 77,276 patients diagnosed with TC between 1974 and 2013, the average age at diagnosis was 48 years old. Meanwhile, another study conducted in Korea, known as the National Epidemiologic Survey of Thyroid Cancer (NEST), reported a mean age of 46.9 years [6,17].

Over recent decades, there has been a significant rise in the number of people who are overweight or obese. A BMI greater than 25 is a well-known indicator of the risk of obesity-related cancers. Many studies have explored the link between obesity and TC, and the meta-analysis conducted by Schmid et al. found that the risk of TC increased by 30% for each five-point rise in BMI [18-19].

A recent review of 31 studies that included over 24 million people found that the risk of TC increases in those who are overweight (RR = 1.26, 95% CI = 1.24-1.28; p <0.001) and does so even more in those who are obese (RR = 1.50, 95% CI = 1.45-1.55; p <0.001). Conversely, being normal or underweight is shown to protect against TC (RR = 0.68, 95% CI = 0.65-0.71; p <0.001 and RR = 0.92, 95% CI = 0.91-0.93; p <0.001, respectively). These findings are consistent with our results, where 20.2% of the study population were classified as overweight and 68.1% obese [20].

The majority of the patients in the study were diagnosed with papillary TC subtypes, while follicular and medullary TC were the least common subtypes, which is consistent with global data [1]. The classic variant of PTC was the most prevalent subtype in the study sample, which contradicts the findings reported by Jung et al. [21], where the follicular variant of PTC was more common. These results suggest that there may be variations in the prevalence of different subtypes of TC across different populations [21-23].

TI-RADS determines the thyroid nodule malignancy risk based on the presence or absence of high-risk sonographic features (hypoechoic, irregular margins, and microcalcifications), allowing for a better selection of thyroid nodules undergoing Fine Needle Aspiration Cytology (FNAC). The Bethesda system established a standardized, category-based reporting system for thyroid FNA specimens consisting of six diagnostic categories. Both facilitate effective communication among cytopathologists, endocrinologists, surgeons, and radiologists [8, 9, 24].

Of the 67 cases categorized as TI-RADS 4 (Moderately suspicious nodules), 12 were benign on FNAC (Bethesda II) and 27 had an FNAC biopsy established as cancer (Bethesda VI). At the same time, the remaining 27 patients fell within Bethesda III, IV, and V. Of the 69 cases categorized as TI-RADS 5 (malignant pattern in the US), 17 cases were suspicious on FNAC (Bethesda V) and 42 cases had an FNAC biopsy established as cancer, giving a 60% concordance between the US and FNAC. Furthermore, 65% of the proven TC (110/169) fell within Bethesda V and VI. The correlation between TI-RADS and Bethesda has been evaluated in previous studies. For example, Horvath et al. reported that TI-RADS 2 carries no risk of malignancy, TI-RADS 3 carries a low (3.4%) risk, TI-RADS 4 has a moderate to high-risk range (10-80%), and TI-RADS 5 has a high risk (87%) [25].

There have been varying results in studies investigating the concordance between the TI-RADS classification and the Bethesda system. For example, one study found a 60% accuracy rate, with 12 out of 20 patients with a TI-RADS-5 having malignant cytology results and the remaining having benign results. However, another study reported a higher accuracy rate of 85.7%, with 90 out of 105 patients with a TI-RADS 5 nodule having an FNAC report of malignancy [8, 24].

In our study, we found that nodules with malignant cytology results (Bethesda VI) fell in either TI-RADS 4 (40%) or TI-RADS 5 (60%). On the other hand, none of TI-RADS 1-3 were classified as Bethesda VI.

LVI remains unaffected by key demographic factors such as age, gender, and tumor size. However, our results differed from those of Sezer et al., who analyzed the relationship between LVI and the clinicopathological features of papillary thyroid carcinoma. Their research showed male gender, larger tumor size, older age, lymph node metastasis, and pathological lymph nodes to all be factors associated with a higher incidence of LVI [26].

Lymph node involvement is rare in patients with follicular thyroid carcinoma; however, in papillary thyroid carcinoma, preoperative neck sonography detects up to 30% of the regional lymph node metastasis. The current study indicated that lymph node metastasis is significantly more prevalent in males and those under 50 and that tumor size does not significantly impact the presence of lymph node metastasis [27]. In their meta-analysis to investigate the potential risks for lymph node metastasis in patients with papillary thyroid carcinoma, Mao et al. found clinical and pathological features such as male gender, younger age (<45 years), tumor size (>1 cm), multifocality, extrathyroidal extension, and capsular invasion to be related to increased risk for lymph node metastasis [28].

The extrathyroidal extension can have significant implications on the treatment and prognosis of patients with DTC, particularly when it comes to deciding whether to administer radioactive iodine. According to the 2015 ATA guidelines, patients with macroscopic extrathyroidal extension are considered high-risk, while those with microscopic extension fall into the intermediate-risk category. Proper classification of the level of extrathyroidal extension is crucial in determining the appropriate management plan and improving patient outcomes [29].

Based on our findings, the presence of extrathyroidal extension is significantly influenced by the tumor subtype, while age, gender, and tumor size have no significant impact. Specifically, the tall-cell and oncocytic variants of papillary TC showed a significantly higher percentage of extrathyroidal extension. This finding contrasts Kuo et al.’s report, which suggested that age, BMI, tumor size, and BRAF mutation are preoperative factors associated with extrathyroidal extension in PTC [30].

Finally, it is important to consider the limitations of this study's findings. Like many studies, the current study's design is not without limitations. Specifically, retrospective studies may have limitations due to their design. These studies rely on chart reviews that were not originally intended for research. It is of utmost importance to take into consideration the patient-specific and tumor-related characteristics when assessing the clinical profile of TC.

## Conclusions

TC, particularly DTC, is rising globally, including in Saudi Arabia. The clinical profile of affected patients varies based on their clinical characteristics such as age, gender, and BMI, as well as the histopathological type of DTC. In the current study, TC mainly affects middle-aged women with a BMI over 25, multiple suspicious ultrasonographic features (TI-RADS 4 or TI-RADS 5), and abnormal FNAC (Bethesda V and VI). It has been observed that male gender and younger age are significant risk factors for lymph node involvement in TC, while tumor size does not seem to affect it. Although no clinical risk factors are associated with extrathyroidal extension, certain histopathological subtypes with aggressive behavior may increase the likelihood of extrathyroidal extension. However, there is conflicting data about the clinical risk factors for the development of TC, lymph node involvement, and extrathyroidal extension. Hence, further research is required to clarify this conflict.
